# The State Medical Treatment of School Children

**Published:** 1909-09-18

**Authors:** 


### The Hospital

A Journal of

The Medical Sciences and Hospital Administration.

New Series. No. 134, Vol. V. [No. 1206, Vol. XLVI.] SATURDAY, SEPTEMBER 18, 1909.

### THE STATE MEDICAL TREATMENT OF SCHOOL CHILDREN.

Once the principle is accepted that the State owes it to the children of the poor, whose education it provides for, to examine into their state of health, it logically must follow, as the night the day, that the State thereby incurs a further obligation towards its elementary scholars. This duty it owes not only to the school children in general, but also, to some extent at least and in certain cases, towards the individual child. Medical treatment, upon some organised plan, is an inevitable corollary to State-directed and State-supported medical inspection of elementary school children. In this respect it differs from the ordinary Public Health Service. The latter presumably can well continue to depute medical treatment, wherever its officers hold this to be necessary, to the general practitioner, to the Poor Law medical officer, or to the hospital—at least until the hypothetical future time when the entire medical profession is reorganised upon Socialistic lines, in some such manner as was indicated in an article contributed to these columns last year, upon the "Socialisation of the Medical Profession."

As was pointed out in the *Times* of September 6, 1909, the State has indeed step by step assumed a parental attitude towards the children of the poorer classes of this country. State medical inspection, and State medical treatment of some sort follow naturally upon the provision by the State of compulsory education. Moreover, the grotesque absurdity of forcing school-work down the throats of children whose most urgent need at the moment is food, has already brought the question of State feeding of elementary school children into the category of difficult and anxious problems of public policy. This of course is not the place for an expression of opinion upon the wisdom or necessity of the assumption by the State of a parental attitude, beset with increasing obligations, towards the children of the million. The State, wisely or unwisely, and in the best or in the worst interests of the lower classes themselves, has already undertaken a position towards the children of the poor, which places it very much *in loco parentis*. Thus we are left to consider at the moment merely the question of how the State, its obligation in this matter already incurred, may best deal with the problem of the State-aided and State-directed medical treatment of its elementary school children. This question has already been referred to in our columns, and we have previously noted the division of expert (and pseudo-expert) opinion into two divergent branches. The one camp advocates, in a word, a compromise between the State and the hospitals; the other desires the establishment of "school clinics," a term which explains itself.

Now the Education Committee of the London County Council, as we remarked at the time, clearly showed by its manner of approaching various London hospitals this year, with a view to an extension of their practice in this direction, that they favoured the less drastic and revolutionary method of treating the physical defects discovered during the medical examination of London elementary school children. They seemed to shrink from the idea of the "school clinic." As was to be expected, their action and their supposed motives gave rise to considerable comment and not a little criticism amongst students of social policy and administration, amongst hospital authorities, and amongst those medical men who are interested in such questions. The British Medical Association, following a discussion of the matter at its recent annual meeting at Belfast—which produced a resolution regretting the line adopted by the London County Council—has now taken action in favour of the "school clinic" idea; and it will be interesting to see what the effect of its public partisanship will be. It should be stated that the County Council since circularising certain London hospitals and publishing an abstract of their replies earlier in the summer has now made arrangements with some of these institutions for the treatment of a few of its youthful charges; and this action forms the basis of the British Medical Association's indictment. The Belfast resolution regretted the action of the London County Council and urged the medical staffs of hospitals to obstruct this threatened abuse of voluntary charity—which moreover provides inadequately for a public evil. The resolution has now been supplemented by an exposition of the grounds upon which it is based, and has, we learn, been forwarded in official form by the British Medical Association to the President of the Board of Education. Copies have also been sent to the authorities, lay and medical, of the hospitals of London, and to the entire medical profession practising within the metropolitan area. Since some organised, far-sighted, economical method of treatment seems inevitable, and in many ways desirable, the "school clinic" system certainly appeals far more to us, as a practical business measure, than does the other. "School clinics" have already, it would seem, established themselves on a sound footing in various parts of Germany—that country of municipal administration and social progress; whereas the method favoured by the L.C.C. Education Committee, however well it might work at first, must surely grow out of hand as time passes and lead to endless difficulties, abuses, and complications. Therefore we await with interest the result of the Association's manifesto, and express ourselves in sympathy with its objects and its main principles.

